# Vitamin D and Parathyroid Hormone Status in Female Garment Workers: A Case-Control Study in Bangladesh

**DOI:** 10.1155/2017/4105375

**Published:** 2017-04-03

**Authors:** Shakil Mahmood, Matiur Rahman, Subrata Kumar Biswas, Shaikh Nazmus Saqueeb, Shiblee Zaman, M. Manirujjaman, Rasheda Perveen, Nurshad Ali

**Affiliations:** ^1^Department of Biochemistry, Bangabandhu Sheikh Mujib Medical University, Shahbag, Dhaka 1000, Bangladesh; ^2^Department of Biochemistry and Molecular Biology, Gonoshasthaya Samaj Vittik Medical College and Hospital, Gono University, Savar, Dhaka 1344, Bangladesh; ^3^Department of Biochemistry and Molecular Biology, Shahjalal University of Science and Technology, Sylhet 3114, Bangladesh

## Abstract

Despite the abundant sunlight, vitamin D deficiency is prevalent in South Asian countries including Bangladesh. Information on vitamin D level is insufficient in adults particularly in female garment workers in Bangladesh. This study was designed to evaluate the status of vitamin D, parathormone (PTH), calcium, and alkaline phosphatase (ALP) among the female garment workers in Bangladesh. Blood samples were collected from female garment workers (*n* = 40, case group) and general female workers (*n* = 40, control group) in Dhaka. Serum vitamin D, PTH, calcium, and ALP were measured by chemiluminescence microparticle immunoassay. The mean level of vitamin D was significantly (*p* < 0.001) lower in case (14.2 ± 2.6 ng/mL) than in the control (22.4 ± 2.4 ng/mL) group. No significant difference was found at mean of PTH and calcium between case (33.9 ± 17.2 pg/mL; 9.1 ± 0.6 mg/dL, resp.) and control (35.9 ± 16.3 pg/mL; 9.3 ± 0.6 mg/dL, resp.) group. The mean ALP in case (117.2 ± 14.4 U/L) group was significantly (*p* < 0.001) higher than the control group (80.5 ± 30.6 U/L). Overall, PTH level did not show significant correlation with vitamin D. However, calcium and ALP levels showed a significant positive (*p* < 0.05) and negative (*p* < 0.001) correlation with vitamin D, respectively. This study indicates a high prevalence of vitamin D deficiency in the female garment workers in Bangladesh.

## 1. Introduction

Vitamin D is a fat soluble steroid hormone which plays an important role in maintaining normal level of calcium and phosphorus in blood. It promotes bone mineralization and is considered as an important determinant of bone health status through absorption of calcium and the secretion of parathyroid hormone (PTH) [1–3]. Natural sunlight exposure is the major source of vitamin D for children and adults [4]. A small number of foods like cod liver oil, sea fish, and egg yolk naturally contain vitamin D or breakfast cereals and milk fortified with vitamin D; thus inadequate exposure to sunlight may causes vitamin D deficiency [4]. Vitamin D deficiency causes rickets, osteomalacia, and other hypovitaminosis D-related disorders like low bone mass, osteoporosis, and fractures and these are a common problem worldwide [5, 6]. Individuals who work indoors or at day with insufficient sunlight exposure may develop vitamin D deficiency. Socioeconomic factors like homebound lifestyle and clothing can affect the sun exposure to individuals [7, 8].

Vitamin D deficiency is prevalent in various parts of the world including South Asia [8]. Bangladesh is a tropical country of South Asia where sunlight is abundant almost year-round; therefore it is hard to believe the prevalence of vitamin D deficiency among the Bangladeshi adults [9]. However, a high prevalence of vitamin D deficiency in Bangladeshi women has been reported in recent studies [8–10].

Bangladesh is well known worldwide for its garments products. A large number of young female workers about 4 million worked in export based garment industries in Bangladesh. The female garment workers are mainly from rural areas with low socioeconomic background and they work typically 10–12 hours/day in an overcrowded and substandard environment [9]. Bangladesh is a predominantly Muslim society where a covered-up style dress is very common among the women in different socioeconomic classes [9]. They are used to homebound lifestyle with little outdoor activity. Moreover, they frequently use sunscreen to prevent their skin from dark color. A high prevalence of subclinical vitamin D deficiency has been reported in veiled Kuwaiti women [11]. Few more studies also reported vitamin D deficiency at various degrees in tropical and subtropical countries among children and women [12–14].

Although a major part of garment workers in Bangladesh are young females, so far, only one study has been conducted to investigate the vitamin D status among them [9]. According to authors [9] opinion, a missing point in their study was the absence of control subjects which can be compared with garment workers to know the actual status of vitamin D level in Bangladeshi female workers. The present study was designed to evaluate the vitamin D and parathyroid hormone status in female garments workers and compare them with female general workers considering same socioeconomic status in Bangladesh.

## 2. Materials and Methods

### 2.1. Study Subjects

This study was conducted in an export-oriented garment factory located in a suburban area belonging to “The Immaculate Textile Ltd.,” Dhamrai, Dhaka, Bangladesh. The garment factory was a multistoried building which maintains a standard quality of working environment for the workers. A total of 80 blood samples were collected in April 2015 from the garment female workers (*n* = 40) as a case group and general female workers (*n* = 40) as a control group. The control study subjects were mainly agricultural and construction workers who worked at sufficient sunlight. They were recruited from the same area matching with age, sex, socioeconomic status, marital status, and educational level as of the garment workers. The subjects with pregnancy, chronic kidney, liver and endocrine diseases, parathyroid, and calcium related diseases or who had taken vitamins and minerals supplements were excluded from the study. The study participants were mainly young women (age 20–40 years) migrated from low-income rural families. They work from morning to evening 6 days per week and cover their body using covered-up style dresses except faces and hands. As sampling plan, first we contacted the Chief Medical Officer at “The Immaculate Textile Ltd.” to explain the aims of the study and asked for their kind cooperation. Then all participants were informed about the study and a written consent was obtained from all of them prior to inclusion in the study. With all aseptic precautions 5 mL of venous blood was drawn from antecubital vein of each participant in a disposable plastic syringe and was delivered immediately into a clean tube, which was kept in standing position till clot formation. The blood samples were centrifuged at 3000 rpm (about 2500 ×g) for 10 min for isolation of serum. The serum samples were then stored at −20°C at the Department of Biochemistry, Bangabandhu Sheikh Mujib Medical University (BSMMU). All participants were asked to report their daily, weekly, and normal eating habits in a short food questionnaire; some individual data (age, height, and body weight) were also recorded in the form. This study received Institutional Review Board approval from BSMMU.

### 2.2. Laboratory Measurements

Serum 25(OH)D was used to evaluate the vitamin D status among the study subjects. The laboratory analysis was carried out at the Department of Biochemistry, BSMMU. Serum concentration of 25(OH)D was measured by chemiluminescence microparticle immunoassay (CI 4100 ARCHITECT, USA). The intra- and interassay coefficients of variation (CV) were 6.5 and 7.4, respectively. The serum parathormone (PTH) was measured by chemiluminescence microparticle immunoassay (CI 4100 ARCHITECT, USA). The intra- and interassay CV for PTH were 6.8 and 5.4, respectively. Moreover, serum levels of alkaline phosphate activity (ALP) and calcium were also analyzed by chemiluminescence microparticle immunoassay (CI 4100 ARCHITECT, USA). The inter- and intra-assay CV for ALP and calcium analyses were less than 7%. Vitamin D deficiency was defined as a serum 25(OH)D level below 20 ng/mL and insufficiency as a serum 25(OH)D level of 21–29 ng/mL recommended by the Institute of Medicine 2011 [4]. Serum PTH > 65 pg/mL was considered as high level in blood (Bosworth et al. 2013).

### 2.3. Statistical Analysis

The statistical analysis was carried out using the software IBM SPSS Statistics version 22. Data are presented as mean, median, and ranges of the parameters. Comparison between the groups was made by independent sample *t*-test. Pearson's correlation coefficient (two-tailed) was used to assess the correlation of variables with age and body mass index (BMI) of the participants. Association of serum 25(OH)D with PTH, ALP, and calcium concentration was done using Pearson's correlation coefficient (two-tailed). One-way ANOVA was used to compare the serum 25(OH)D concentrations in the food consumption groups. A level of alpha 0.05 was assigned for statistical significance.

## 3. Results

### 3.1. Demographic Characteristics of the Participants

In Dhamrai region of Dhaka district, the number of case and control female study subjects was equal. Their mean age was 27.2 ± 5.5 and 28.3 ± 4.8 years in case and control group, respectively. The average body mass index (BMI) was 22.2 ± 2.5 and 22.9 ± 2.2 kg/m^2^ in the groups, respectively. Overall, there was no significant difference for age, BMI, and waist circumference (WC) between the groups which reflected the homogeneity of the groups. Details on baseline characteristics of both cohorts are given in Table 1.

### 3.2. Serum Concentration of 25(OH)D

The serum concentration of 25(OH)D reflects the nutritional status of vitamin D. The average level of serum 25(OH)D was significantly higher (*p* < 0.001) in control (22.4 ± 2.4 ng/mL) than the case (14.2 ± 2.6 ng/mL) group (Table 3). When serum 25(OH)D were categorized as deficient, insufficient and sufficient subgroup, 100% and 17% of the study subjects were 25(OH)D deficient in case and control group, respectively. In control group, 80% subjects were in insufficient and only 3% subjects were in sufficient subgroup (Table 2).

### 3.3. Serum Concentration of PTH, Calcium, and ALP and Their Correlation with 25(OH)D

**Figure 1:**
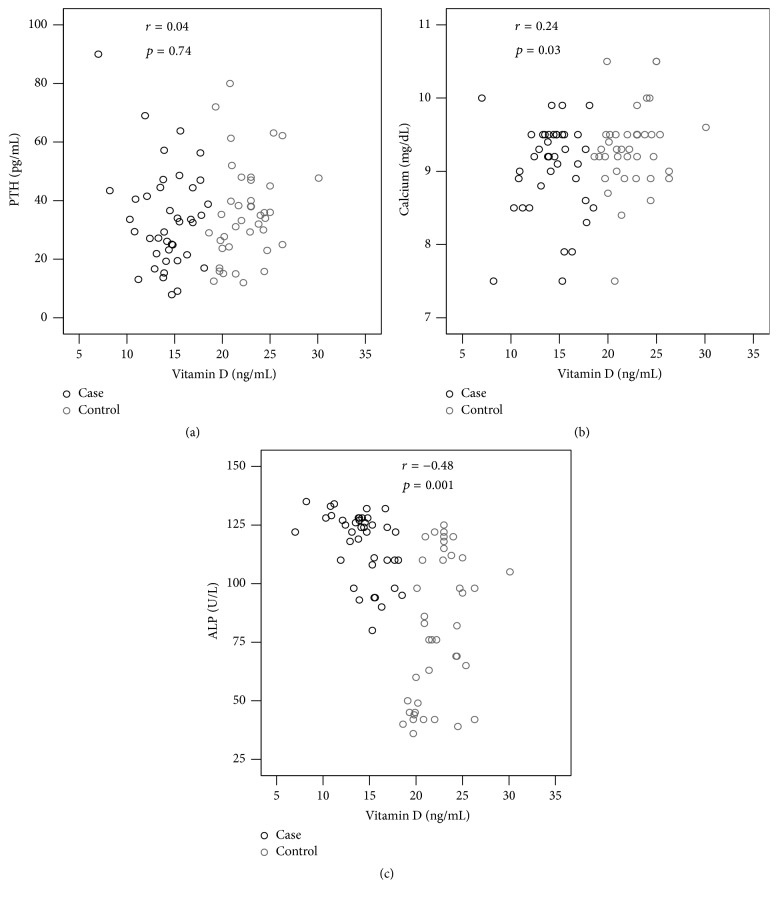
Correlation of serum concentration of vitamin D [25(OH)D] with PTH (a), calcium (b), and ALP (c).

There was no significant difference at the mean level of calcium and PTH between case (9.1 ± 0.6 mg/dL; 33.9 ± 17.2 pg/mL, resp.) and control (9.3 ± 0.6 mg/dL; 35.9 ± 16.3 pg/mL, resp.) group (Table 3). The mean concentration of serum ALP in case (117.2 ± 14.4 U/L) group was significantly (*p* < 0.001) higher compared to control (80.5 ± 30.5 U/L) group. Serum PTH level did not show significant correlation with vitamin D; however, calcium and ALP levels showed a significant positive (*p* < 0.05) and negative (*p* < 0.001) correlation with vitamin D, respectively (Figure 1).

### 3.4. Food Intake and Serum Levels of 25(OH)D

Based on information provided in the short food frequency questionnaires (FFQ), possible correlations were analyzed between serum 25(OH)D and food consumption in the cohorts. Serum 25(OH)D level was slightly high in more egg and fish consumption group. However, no significant difference was found within the groups (Table 4).

## 4. Discussion

Vitamin D deficiency is prevalent in several parts of the world, but relatively a few studies have been carried out to investigate the vitamin D status in working populations. In Bangladesh, there is an insufficient information about vitamin D [25(OH)D] level in indoor female workers. The present study results indicate a high prevalence of vitamin D deficiency in female garment workers (100%) and low prevalence in female general workers (17%) in Bangladesh (Table 2).

In present study, we noticed that the garment workers spent a major part of the day time (10–12 h/d) in the office building. In general, they have a regular sunshine exposure (5–15 min/d) in the very early morning on their way from living place to their working places [9]. In summer 10–15 min outdoors sun exposure, two to three times in a week, might be sufficient for effective vitamin D production in the skin [15]. However, the present investigation indicates that short time sunshine exposure may not be effective enough for vitamin D synthesis in skin in the Bangladeshi female workers, which has also been reported in previous study by Islam et al. 2008. In our study, the control subjects were mainly construction workers who are exposed to sunshine for longer time because of their outdoor working environment when compared to the garment workers. In spite of abundant sun exposure, only 3% of the study subjects had the sufficient level of vitamin D [25(OH)D].

The prevalence of vitamin D deficiency has been reported in different degrees in several countries in Asia. Low level of vitamin D has been reported in the adult population of Malaysia [16], China [17], Thailand [18], Korea [19], and India [20, 21]. A study in Australia reported the high prevalence of vitamin D deficiency among the Australian Muslim healthy women [22]. Vitamin D containing food like high intake of fish, fortification of food with vitamin D, or supplementation of vitamin D may be the causes of these differences.

In present investigation, four in 80 participants had hyperparathyroidism (serum PTH > 65 pg/mL), which could not explain why serum PTH was increased in participants with low level of serum vitamin D [25(OH)D]. The present study findings comply with the report that low level of vitamin D does not always increase the serum PTH level [2]. Our study results slightly differ from the previous study in Bangladesh where only one in 200 participants had hyperparathyroidism [9]. Substantial studies suggest that serum levels of vitamin D [25(OH)D] concentration from 12 to 50 ng/mL are required to maintain the normal serum PTH level in individuals [2, 23–25]. An inverse correlation between serum vitamin D and PTH concentration has been reported elsewhere [26]. In present investigation only four in 80 participants had the serum PTH level above 65 pg/mL and it was observed that serum PTH began to increase when serum 25(OH)D was lower than 20 ng/mL. This inverse relationship between serum 25(OH)D and serum PTH indicates hypovitaminosis D in the study subjects, although there is less information on specific cut-off value for serum vitamin D [25(OH)D] deficiency and insufficiency which can demark the level between sufficiency and insufficiency [9]. According to the recommendation of Institute of Medicine (IOM), vitamin D deficiency has been defined as a 25(OH)D of less than 20 ng/mL and insufficiency as a 25(OH)D of 21–29 ng/mL [27–33]. Interestingly, we found only one subject who had the 25(OH)D level above 29 ng/mL in control group. However, overall no significant relationship was observed between serum vitamin D and serum PTH which supports the finding reported by Elsammak et al. 2011. Therefore, serum concentration of PTH may not be used clinically as an indicator of vitamin D deficiency and regardless of calcium and PTH results, serum 25(OH)D should be measured if vitamin D deficiency or insufficiency is predicted [34].

In addition, a higher level of ALP was found in the study subjects and it was higher in case than the control group (Table 3). Our findings are in line with the previous studies conducted on veiled and nonveiled Bangladeshi women [8]. High PTH level is a usual finding in osteomalacia. The negative correlation (*p* < 0.001; Figure 1) of serum vitamin D with serum ALP in the study subjects indicating an osteomalacic effect on bone and the higher level of total alkaline phosphatase (ALP) activity in female garment workers indicated that bone turnover was increased in these groups. However, higher level of serum ALP activity probably reflected bone isoenzymes because of lower levels of serum 25(OH)D in garment workers.

In the study, we found that the mean concentration of serum calcium was relatively low in case compared to control group. A lower level of calcium in garment workers has also been reported in the previous study [9]. Vitamin D deficiency results in a decrease in intestinal calcium absorption, resulting in a decline in calcium concentrations in the serum. Calcium deficiency increases the catabolism of 25(OH)D in the liver and thereby increases the requirement of vitamin D [35].

The time of year is an important factor in measurement of vitamin D levels in the diagnosis of insufficiency or deficiency. A previous study found that summer is the ideal time to measure vitamin D levels as there is seasonal variation with a 14% increase of 25(OH)D concentrations in men in summer [36]. We measured the serum concentration of 25(OH)D in the summer season, which could have been the highest level. In fact, a comparison of our obtained data with other studies may not be entirely appropriate as study cohorts; methods and sampling period varied from the present analysis.

In our study, intake of selected vitamin D containing food did not show significant correlation with vitamin D. A similar finding was also found in Norris [37] investigation where authors suggested that about 10% of vitamin D is derived from dietary sources. Holick and Chen [38] indicated that dietary intake of the vitamin D is a relatively poor predictor of overall vitamin D status. Insignificant association between dietary vitamin D intake and plasma level of vitamin D 25(OH)D has been reported in studies conducted in Europe [39–41]. The exact reasons for the vitamin D deficiency in Bangladeshi women are unclear. We presume and agree with the authors [9] who indicated that low intensity of sunshine, air pollution, using of sunscreen, and covered-up style dresses as well as dark skin might be the possible reasons for inadequate vitamin D synthesis in the skin in the Bangladeshi women.

## 5. Conclusion

The present investigation evaluates the serum vitamin D status in female garment workers as well as female general workers in Bangladesh. The results indicate a high prevalence of vitamin D deficiency in the Bangladeshi female garment workers (100%) which could be a serious concern. Therefore, both governmental and nongovernmental health professional and health policy maker should pay attention to increase the awareness on vitamin D deficiency and its adverse effects on health especially in adult female garment workers in Bangladesh. A comprehensive programme including extensive awareness of the importance of sunlight exposure and improved dietary supplies of calcium and vitamin D as well as inclusion of food fortification is recommended to prevent the vitamin D deficiency in Bangladeshi women.

## Figures and Tables

**Table 1:** Baseline demographic characteristics of study subjects.

Characteristics	All	Case	Control	*p* value
	(*n* = 80)	(*n* = 40)	(*n* = 40)	
Age (year)	27.8 ± 5.1	27.2 ± 5.5	28.3 ± 4.6	0.32
Weight (Kg)	52.2 ± 5.2	50.9 ± 5.6	53.5 ± 4.4	0.03
Height (cm)	152.1 ± 5.1	151.3 ± 5.1	152.9 ± 4.9	0.78
BMI (Kg/m^2^)	22.6 ± 2.4	22.2 ± 2.5	22.9 ± 2.2	0.23
Waist circumference (cm)	72.1 ± 6.2	72.6 ± 6.6	71.6 ± 5.8	0.51
Hip circumference (cm)	81.3 ± 5.4	81.9 ± 5.9	80.8 ± 4.8	0.38
Education level				
≤primary	30	20	10	0.68
Primary-junior level	30	10	20	
≤secondary	20	10	10	

Values are presented as mean ± SD. Independent sample *t*-test was done to find out the level of significance between the groups.

**Table 2:** Categorization of study subjects depending upon vitamin D [25(OH)D] status.

Vitamin D	Case	Control
	*n* (%)	*n* (%)
Deficient (<20 ng/mL)	40 (100)	7 (17)
Insufficient (21–29 ng/mL)	0	32 (80)
Sufficient (≥30 ng/mL)	0	1 (3)

Serum vitamin D [25(OH)D] concentration was categorized according to Institute of Medicine (IOM) 2011.

**Table 3:** Serum concentration of vitamin D [25(OH)D], PTH, calcium, and ALP in the study subjects.

Variables	Case (*n* = 40)		Control (*n* = 40)		*p* value
	Mean ± SD	Median (range)	Mean ± SD	Median (range)	
25(OH)D (ng/mL)	14.2 ± 2.6	14.3 (7.0–18.5)	22.4 ± 2.4	22.1 (18.6–30.1)	<0.001
PTH (pg/mL)	33.9 ± 17.2	32.6 (7.9–90.0)	35.9 ± 16.3	34.5 (12.0–80.0)	0.609
Calcium (mg/dL)	9.1 ± 0.6	9.2 (7.5–10.0)	9.3 ± 0.6	9.3 (7.5–10.5)	0.057
ALP (U/L)	117.2 ± 14.4	123.0 (80.0–135.0)	80.5 ± 30.6	79.0 (36.0–125.0)	<0.001

*p* values obtained by comparing the mean concentration of variables of case and control group. Independent sample *t*-test was used to evaluate the results.

**Table 4:** Intake of vitamin D containing selected food and vitamin D levels among the study subjects.

Food items	Case		Control		*p* value
	*n*	Mean ± SD	*n*	Mean ± SD	
Egg consumption					
Not at all	5	12.4 ± 2.2	2	21.4 ± 2.1	0.152
1–4 times/month	15	13.5 ± 2.4	10	22.1 ± 1.9	
1–4 times/weekly	10	14.9 ± 3.1	15	22.6 ± 2.3	
1-2 times/day	10	15.4 ± 2.6	13	23.1 ± 3.4	
Fish consumption					
Less than 3 times/month	10	13.9 ± 2.2	3	21.8 ± 3.2	0.459
1–6 times/week	20	14.3 ± 2.6	25	22.4 ± 2.2	
1-2 times/day	10	14.5 ± 2.9	12	22.7 ± 2.2	

One-way ANOVA was used to find out the level of significance.
